# Herbal tea, a novel adjuvant therapy for treating type 2 diabetes mellitus: A review

**DOI:** 10.3389/fphar.2022.982387

**Published:** 2022-09-30

**Authors:** Xiangyuan Zhang, Lili Zhang, Boxun Zhang, Ke Liu, Jun Sun, Qingwei Li, Linhua Zhao

**Affiliations:** ^1^ Department of Institute of Metabolic Diseases, Guang’anmen Hospital, China Academy of Chinese Medical Sciences, Beijing, China; ^2^ Graduate College, Beijing University of Traditional Chinese Medicine, Beijing, China; ^3^ Graduate College, Changchun University of Traditional Chinese Medicine, Jilin, China

**Keywords:** herbal tea, type 2 diabetes, randomized controlled trial, review, mechanism

## Abstract

Type 2 diabetes mellitus (T2DM) is a metabolic, endocrine disease characterized by persistent hyperglycemia. Several studies have shown that herbal tea improves glucose metabolism disorders in patients with T2DM. This study summarizes the published randomized controlled trials (RCTs) on herbal tea as a adjuvant therapy for treating T2DM and found that herbal teas have potential add-on effects in lowering blood glucose levels. In addition, we discussed the polyphenol contents in common herbal teas and their possible adverse effects. To better guide the application of herbal teas, we further summarized the hypoglycemic mechanisms of common herbal teas, which mainly involve: 1) improving insulin resistance, 2) protecting islet β-cells, 3) anti-inflammation and anti-oxidation, 4) inhibition of glucose absorption, and 5) suppression of gluconeogenesis. In conclusion, herbal tea, as a novel adjuvant therapy for treating T2DM, has the potential for further in-depth research and product development.

## 1 Introduction

Diabetes mellitus (DM) poses a major threat to global health. According to the latest report of International Diabetes, by 2045, approximately 783 million people worldwide will suffer from DM, of which more than 90% will have type 2 diabetes mellitus (T2DM) (Zheng et al., 2018a; International Diabetes Federation, 2021). In the early stages of T2DM, only a simple increase in blood sugar levels was observed. As the disease progresses, it can lead to serious complications such as diabetic retinopathy, diabetic kidney disease, and diabetic foot ulcers, which can cause great pain and a heavy economic burden to the patient (Harding et al., 2019). Clinical trials have shown that improved blood glucose control can slow the progression of T2DM and its complications (Taylor et al., 2021). Researchers have developed insulin, metformin, and other drugs that function through different mechanisms to lower blood sugar levels. However, patients with T2DM experience difficulties controlling their blood glucose levels, such as high blood glucose fluctuations, susceptibility to hypoglycemia, and a high incidence of adverse gastrointestinal reactions (Wilson and Castle, 2020; Guo et al., 2021). Therefore, it is necessary to develop a safe and effective auxiliary treatment for T2DM.

For thousands of years, herbs have been widely used to prevent and treat various diseases owing to their remarkable efficacy and minor side effects (Jiang et al., 2013; Huang et al., 2018a; Jeon et al., 2019). With the popularization of tea drinking and the gradual accumulation of medical practice experience, some herbs with remarkable curative effects are processed and directly brewed for drinking, forming a new dosage form called herbal tea (HT). HT is a combination of herbs and traditional teas. Generally, HT is defined as water infusions/decoctions prepared with herbal ingredients other than *Camellia sinensis* (L.) Kuntze (Fu et al., 2018). It is prepared from different herbs’ seeds, fruits, flowers, leaves, stems, and roots. HT is widely used as a traditional medical treatment for T2DM. In the Tang Dynasty of China, doctors created an HT called “Xi Thirst Tea” to improve symptoms such as urine sugar, polydipsia, and polyphagia. In South Africa, Rooibos Herbal Tea and Honeybush Herbal tea are considered attractive strategies for managing T2DM and have been successfully commercialized (Ajuwon et al., 2018). While some clinical trials have confirmed the beneficial effects of HTs in controlling blood glucose levels in recent years, the quality of these studies remains unclear. In addition, the intake and habits of HT consumption vary among individuals, and the bioavailability of compounds in HTs, such as polyphenols, polysaccharides, and alkaloids, is susceptible to various chemical and biological factors. Therefore, a more comprehensive understanding of HTs’ hypoglycemic mechanisms should help prevent diabetes and its related complications. While reviews on the mechanisms by which HTs or herbs improve blood glucose levels have been published in recent years, these reviews either fail to include HTs commonly used worldwide or comprehensively discuss the hypoglycemic mechanisms of important herbs in HTs, or fail to summarize the latest findings. To address these limitations, we compiled the evidence and level of evidence for the clinical efficacy of HTs in treating T2DM and provided a comprehensive discussion of the glucose-lowering mechanisms of HTs and their bioavailability.

## 2 Clinical research

We searched electronic databases including PubMed, Cochrane Library, Embase, WOS, CNKI, CBM, CSPD, and VIP for variants of the terms “herbal tea,” “herbal medicine,” and “beverage,” “type 2 diabetes mellitus,” or “non-insulin-dependent diabetes.” In addition, clinical trials were searched to identify relevant unpublished data. We included clinical studies that met the following criteria: study participants were diagnosed with T2DM and randomly assigned to receive HTs or placebo treatment. We also evaluated the quality of the RCTs according to the modified Jadad rating scale (Table 1).

**TABLE 1:** Effectiveness of HTs in the management of T2DM.

Trials	Herbal Tea name	No of patients		Sex(male/female)		Age(years)		Treatment		Treament time
		text group	Control group	text group	Control group	text group	Control group	text group	Control group		
Xie et al. (2020)	Mulberry leaf tea	40	40	26/14	24/16	53.88 ± 14.31	54.08 ± 12.74	Conventional treatment + Metformin sustained-release tablets 0.5 g/Bid + Mulberry tea	Conventional treatment + Metformin sustained-release tablets 0.5 g/Bid	3 months	12 weeks
Yang et al., (2019)	Mulberry leaf tea	50	50	24/26	27/23	43.20 ± 10.72	43.58 ± 10.12	Conventional treatment + Mulberry leaf Black Tea	Conventional treatment	6 months	24 weeks
Rafraf M et al., (2015)	Chamomile tea	32	32	6/26	6/26	50.19 ± 7.08	51.97 ± 6.42	Chamomile Tea	Warm water	2 months	8 weeks
Kaseb et al., (2018)	Chamomile tea	25	25	-	-	55.33 ± 7.85	55.22 ± 6.72	Chamomile Tea	Conventional treatment	1 month	4 weeks
Huyen et al., (2011)	Gynostemma tea	12	12	8/4	9/3	63.5 ± 6.5	57.2 ± 8.2	Gynostemma pentaphyllum Tea	Placebo	3 months	12 weeks
Klein et al., (2011)	Yerba mate tea	11	9	1/10	4/5	54.3 ± 6.9	60 ± 6.7	Mate tea	Dietary intervention	2 months	60 days
Jayawardena et al. (2005)	Kothala himbutu tea	28	23	16/12	12/11	53.2 ± 7.5	54.3 ± 6.9	Kothala Himbutu Tea	placebo	3 months	12 weeks
Jia et al. (2002)	Puda tea	38	32	12/26	9/23	53.77 ± 9.18	58.97 ± 9.07	Puda Tea	Placebo	1 month	4 weeks
CampbellTofte et al. (2011)	Rauvolfia-Citrus tea	12	11	6/5	7/5	63.4 ± 8.8	63.9 ± 5.2	Rauvolfia-Citrus Tea	Placebo	4 months	16 weeks
Yue et al., 2018	Tuckwheat yuzhu tea	53	52	-	-	76.63 ± 7.01	76.63 ± 7.01	Conventional treatment + Tartary Buckwheat Polygonatum Tea	Conventional treatment	6 months	24 weeks
Liu et al., (2018)	Mulberry black tea	20	20	-	-	-	-	Metformin tablets +Compound Danshen Dripping Pills +Mulberry Black Tea	Metformin tablets +Compound Danshen Dripping Pills	3 months	12 weeks
Fang (2001)	Shuning tea	18	15	8/10	6/9	58.3	57.4	Metformin tablets + Shu Ning Tea	Metformin tablets	1 month	4 weeks
Han et al., (2015)	Zhongyue jiangsangao tea	30	30	17/13	16/14	44.8 ± 8.4	45.1 ± 7.9	Metformin tablets + Yuejiang Sangao Herbal Tea	Metformin tablets	3 months	12 weeks
Hu et al., (2018)	Tianhan diabetes tea	52	52	13/39	15/37	56.71 ± 7.52	54.20 ± 6.00	Tianhan diabetes Tea	Blank control	2 months	8 weeks
Mahmoud F et al., 2016	Diabetes tea	30	20	-	-	51.5	54.2	Diabete Tea extract	Placebo	3 months	12 weeks
Wen et al., (2018)	Self-developed herbal tea	50	50	12/38	16/34	67.26 ± 7.10	65.81 ± 6.58	Acupoint self-massage + Herbal Tea	Acupoint self-massage	6 months	24 weeks
Xie et al. (2020)	Mulberry leaf tea	6.74 ± 1.22	6.93 ± 1.60	6.86 ± 0.96	6.87 ± 1.11	9.49 ± 3.06	9.79 ± 2.95	7.83 ± 3.74	9.76 ± 3.72	2.45 ± 1.43	3.19 ± 1.66	3
Yang et al., (2019)	Mulberry leaf tea	5.73 ± 1.15	6.52 ± 1.34	6.05 ± 0.67	6.31 ± 0.58	7.29 ± 1.16	8.96 ± 1.25	-	-	-	-	4
Rafraf M et al., (2015)	Chamomile tea	8.89 ± 3.7	8.74 ± 1.56	7.48 ± 1.59	7.50 ± 0.92	-	-	-	-	4.24 ± 1.95	5.55 ± 1.12	4
Kaseb et al., (2018)	Chamomile tea	8.16 ± 0.37	9.05 ± 0.37	-	-	12.35 ± 0.61	13.20 ± 0.61	-	-	-	-	4
Huyen et al., (2011)	Gynostemma tea	7.0 ± 1.4	8.7 ± 2.2	7.4 ± 1.0	8.1 ± 1.3	-	-	-	-	4.82 ± 2.64	6.89 ± 3.8	7
Klein et al., (2011)	Yerba mate tea	-	-	-	-	-	-	-	-	-	-	2
Jayawardena et al. (2005)	Kothala himbutu tea	-	-	6.29 ± 1.02	6.65 ± 1.04	-	-	-	-	-	-	6
Jia et al. (2002)	Puda tea	9.16 ± 3.66	12.14 ± 3.23	-	-	14.4 ± 13.41	20.01 ± 4.52	8.70 ± 5.09	8.50 ± 4.60	-	-	3
CampbellTofte et al. (2011)	Rauvolfia-Citrus tea	7.7 ± 2.2	8.1 ± 2.1	6.1 ± 1.2	6.7 ± 1.1	14.5 ± 5.5	15.8 ± 3.8	11 ± 4	7 ± 3	-	-	7
Yue et al., 2018	Tuckwheat yuzhu tea	7.71 ± 2.01	8.52 ± 2.32	8.12 ± 1.71	8.63 ± 1.62	-	-	-	-	-	-	4
Liu et al., (2018)	Mulberry black tea	6.47 ± 1.09	7.31 ± 1.42	6.01 ± 0.58	6.42 ± 0.65	8.21 ± 1.34	9.13 ± 1.50	-	-	-	-	3
Fang 2001	Shuning tea	8.0 ± 1.7	9.5 ± 1.9	-	-	9.3 ± 2.6	10.1 ± 2.3	-	-	-	-	2
Han et al., (2015)	Zhongyue jiangsangao tea	7.2 ± 2.3	9.8 ± 2.9	-	-	9.8 ± 2.1	12.4 ± 2.7	7.2 ± 1.4	9.2 ± 1.4	3.2 ± 1.0	4.3 ± 1.5	1
Hu et al., (2018)	Tianhan diabetes tea	6.38 ± 2.25	8.12 ± 3.25	6.21 ± 0.93	6.08 ± 1.14	9.96 ± 2.55	11.25 ± 3.08	-	-	-	-	1
Mahmoud F et al., 2016	Diabetes tea	8.7 ± 0.38	8.12 ± 57	8.26 ± 0.21	7.70 ± 0.37	-	-	-	-	-	-	3
Wen et al., (2018)	Self-developed herbal tea	7.12 ± 0.35	7.02 ± 1.02	6.12 ± 0.35	7.02 ± 1.02	10.12 ± 0.35	11.02 ± 1.02	-	-	-	-	1

There are many types of HTs. Based on the number of medicinal herbs, we divided them into two categories. The first category consists of a single herb. HTs that use one herb as the main ingredient to lower blood sugar levels and are supplemented with other herbs fall into this category. The second category consists of two or more herbs. In this category, it is impossible to determine which herb is the main ingredient responsible for lowering sugar levels. In addition, we summarized the composition, dose, and mode of administration of HTs (Tables 2 and 3).

**TABLE 2:** Herbs in HTs for the treatment of T2DM.

	Herbal tea name	Composition	Parts
Single Herbal Tea	Mulberry leaf tea	Morus alba L.	leaf
	Chamomile tea	*Chamaemelum nobile L.*	flower
	Gynostemma pentaphyllum tea	Gynostemma pentaphyllum (Thunb.) Makino.	leaf
	Yerba mate tea	Ilex paraguariensis A.St.-Hil.	leaf
	Kothala himbutu tea	Salacia renetulata Wight.	root and stem
	Puda tea	Momordica charantia L.	fruit
Compound Herbal Tea	Rauvolfia-Citrus tea	Rauvolfia vomitoria Wennberg	foliage
		Citrus × aurantium L.	fruit
	Tuckwheat yuzhu tea	Fagopyrum tataricum (L.) Gaertn.	seed
		Polygonatum odoratum (Mill.) Druce.	rhizome
	Mulberry leaf black tea	Morus alba L.	leaf
		Camellia sinensis (L.) Kuntze	leaf and bud
	Shunning tea	Morus alba L.	leaf
		Other composition unknown	-
	Zhongyue Jiangsangao herbal tea	Crataegus pinnatifida Bunge	fruit
		*Cassia* obtusifolia L.	seed
		Sophora japonica L.	flower and alabastrum
		Lycium barbarum L.	fruit
		Polygonatum odoratum (Mill.) Druce.	rhizome
		Pueraria lobata (Willd.) Ohwi	root
		Ziziphus jujuba Mill. var. spinosa (Bunge) Hu ex H. F. Chou	seed
		*Dioscorea* opposita Thunb.	rhizome
		Prunus mume (Sieb.) Sieb.et Zucc.	fruit
		*Glycyrrhiza* uralensis Fisch.	root and rhizome
	Tianhan Xiaoke tea	*Astragalus* membranaceus (Fisch.) Bge.	root
		Cornus officinalis Sieb. et Zucc.	fruit
		Ophiopogon japonicus (Thunb.) Ker Gawl.	rhizome
		Rehmannia glutinosa (Gaertn.) DC.	rhizome
		*Dioscorea* opposita Thunb.	rhizome
		Poria cocos (Schw.) Wolf	sclerotium
	Diabetea tea	*Cassia* tora L.	leaf and seed
		*Ficus* racemosa L.	bark and fruit
		Syzygium cumini (L.) Skeels	bark
		*Terminalia* arjuna (Roxb. ex DC.) Wight & Arn.	bark
		*Terminalia* chebula Retz.	fruit
		*Terminalia* bellirica (Gaertn.) Roxb.	fruit
		*Phyllanthus* emblica L.	fruit
		Tribulus terrestris L.	fruit
		Trigonella foenum- graecum L.	seed
		Cardiospermum halicacabum L.	leaf
		Cinnamomum zeylanicum Blume	bark
		Camellia sinensis (L.) Kuntze	leaf
		Azadirachta indica A.Juss.	leafs and seed
		*Ficus* benghalensis L.	bark and fruit
	Self-developed herbal tea	Morus alba L.	leaf
		Crataegus pinnatifida f. major (N.E.Br.) W.Lee	fruit
		Nelumbo nucifera Gaertn.	leaf
		Camellia sinensis (L.) Kuntze	leaf and bud

**TABLE 3:** Dosage and usage of HTs.

Trials	Herbal tea	Single dose (g)/time	Water for making herbal tea	Brewing time	Daily times	Total dosage (g)/day	Treament time	Others
Xie et al. (2020)	Mulberry leaf tea	6	Hot water		2	12	3 months	
Yang et al., (2019)	Chamomile tea	5	Hot water (85–95°F)	-	2	10	6 months	-
Rafraf M et al., (2015)	Chamomile tea	10	100 ml Boiling water	10 min	2	20	2 months	Before lunch and dinner
Kaseb et al., (2018)	Gynostemma tea	9	150 ml Hot water	10 min	3	27	1 month	Drink immediately after meals
Huyen et al., (2011)	Mate tea	3	-	-	2	6	3 months	-
Klein et al., (2011)	Kothala himbutu tea	6.6	330 ml Boiling water	10 min	3	19.8	2 months	Before or during meals
Jayawardena et al. (2005)	Puda tea	-	-	-	3	-	3 months	During meals
Jia et al. (2002)	Rauvolfia-Citrus tea	-	-	-	-	4	1 month	Daily way of drinking tea
Yue et al. (2018)	Tuckwheat yuzhu tea	8	300 ml Hot water		2	-	6 months	After meals
Liu et al., (2018)		30	500 ml Water		at least 3	-	3 months	-
Fang 2001	Shuning tea	-	-	-	3	-	1 month	-
Han et al., (2015)	Zhongyue jiangsangao tea	5–10	Boiling water	Until the tea broth is tasteless		-	3 months	-
Hu et al., (2018)	Tianhan diabetes tea	1.8	-	-	2	-	2 months	-
Mahmoud F et al., 2016	Diabetes tea	2.72	250 ml Boiling water	5 min	at least 4	-	3 months	-
Wen et al., (2018)	Self-developed herbal tea	60	Boiling water			-	6 months	

### 2.1 Herbal teas consisting of a single herb as the main hypoglycemic effect

#### 2.1.1 Mulberry leaf tea

Mulberry leaf (ML) tea consists of dried leaves of *Morus alba* L. and is popular in East Asian countries, such as China and Japan. In traditional Chinese medicine, mulberry leaves are an essential herb for treating diabetes. A 3-months RCT showed that combining ML tea (6 g/d) with metformin could decrease the blood glucose indices; however, there was no significant difference between the two groups (Xie et al., 2020). In comparison, other RCTs using a daily dose of 10 g and 6 months of intervention showed that ML tea could produce more obvious benefits in improving fasting plasma glucose (FPG), glycated hemoglobin (HbA1c), and 2-h postprandial blood glucose (2hPBG) values in patients with T2DM and pre-diabetes, and the incidence of adverse reactions in the intervention group (6.00%) was lower than that in the control group (8.00%) (Yang and Fang, 2019).

#### 2.1.2 Chamomile tea

Chamomile tea is made from dried flowers of *Chamaemelum nobile* L. It is one of the most widely used HTs worldwide, originating from Europe and West Asia (Khan et al., 2014). In a 4-weeks clinical trial, T2DM patients were administered chamomile tea (10 g/100 ml) twice daily before meals. At the end of the experiment, FPG and 2hPBG of T2DM patients were significantly reduced (Bracesco et al., 2011). In another 8-weeks clinical trial, T2DM patients were treated with chamomile tea (3 g/150 ml) after three daily meals. Chamomile tea not only significantly reduced HbA1c concentration, serum insulin level, and homeostatic model evaluation of insulin resistance in T2DM patients but also significantly increased total antioxidant capacity and the activities of superoxide dismutase (SOD), glutathione peroxidase (GSH-Px), and catalase (CAT) in the patients. Therefore, the hypoglycemic effect of chamomile tea may be associated with an improvement in oxidative stress (Zemestani et al., 2016). In addition, the results of both studies suggest that short- or long-term use of chamomile tea can improve blood sugar levels in patients with diabetes. However, the time taken for chamomile tea consumption should not be limited to before or after meals.

#### 2.1.3 Gynostemma tea

Gynostemma tea is made from dried leaves of *Gynostemma pentaphyllum* (Thunb.) Makino. Gynostemma tea has been regarded as an important health medicine in Asian countries, such as China. Based on its remarkable hypoglycemic and antihypertensive properties, GP was also listed as the first batch of “precious traditional Chinese medicine” developed by the “Spark Plan” developed by China’s Ministry of Science and Technology (Su et al., 2021). In an RCT, Gynostemma tea improved blood sugar levels in patients with T2DM. All T2DM patients were randomly assigned to either Gynostemma tea or placebo tea (6 g/day) groups. After 12 weeks of treatment, the FPG level and homeostatic model assessment of insulin resistance (HOMA-IR) were significantly lower in the treatment group than in the control group (Huyen et al., 2010). This study is the first to confirm that Gynostemma tea has a significant hypoglycemic effect and can improve insulin sensitivity. In addition, in this study, Gynostemma tea had no adverse effects, such as liver and renal toxicity. Therefore, it is considered a safe HT.

#### 2.1.4 Yerba mate tea

Yerba mate (YM) tea is made from dried leaves of *Ilex paraguariensis* A. St.-Hil. It is a widely consumed HT in southern Latin American countries, including southern Brazil, Argentina, Paraguay, and Uruguay. In addition, it is rapidly seeping into the global market, including the United States (Heck and de Mejia, 2007). The latest RCT compared the differences between HT and dietary interventions in improving blood sugar levels. A specific measure of dietary intervention is to reduce calorie intake. In this study, all patients were randomly divided into three groups: YM tea, diet intervention, and YM tea plus diet intervention. Specifically, each person had to drink YM tea (330 ml) three times a day and/or receive dietary nutrition counseling for more than 60 days. At the end of treatment, compared with baseline values, only FPG and HbA1c levels in the experimental group decreased significantly (Klein et al., 2011). Therefore, YM can be considered a dietary alternative to control T2DM and pre-diabetes blood sugar levels.

#### 2.1.5 Kothala himbutu tea

Kothala himbutu (KH) tea is a mixture of herbs, and the main ingredients are the root and stem of *Salacia renetulata* Wight (Kishino et al., 2009). In traditional Ayurvedic medicine in India and Sri Lanka, KH is considered a specific treatment for early diabetes (Im et al., 2009). An RCT showed that KH tea significantly reduced HbA1C levels in patients in the intervention group. Furthermore, there were no serious adverse reactions or abnormalities in liver or kidney function (Jayawardena et al., 2005). Therefore, KH tea can be considered effective and safe for reducing blood sugar levels.

#### 2.1.6 Puda tea

Puda tea contains the fruit of *Momordica charantia* L., which is the main ingredient. It is also supplemented with other medicinal plants and traditional tea leaves. Bitter gourd is one of the most widely studied traditional medical drugs (Ooi et al., 2012). In traditional Chinese medicine, Ayurvedic and other traditional medicines are often used in diabetes management. The results of an RCT showed that Puda tea could significantly reduce FPG in patients with T2DM and effectively improve impaired glucose tolerance (Jia et al., 2002). In addition, it can improve symptoms such as polydipsia and polyphagia.

### 2.2 Herbal teas that contain various herbs with a hypoglycemic role

#### 2.2.1 *Rauvolfia-Citrus* tea

*Rauvolfia-Citrus* tea is made with the foliage of *Rauvolfia vomitoria* Wennberg and the fruits of *Citrus aurantium* L. In northern Nigeria, it is believed that taking this HT while giving up alcohol and following a calorie-restricted diet can improve diabetes. In a 4-months RCT, compared with the baseline values before treatment, treatment with RC tea reduced HbA1b and FPG in T2DM patients by 6% and 10%, respectively. In addition, no adverse reactions occurred in any patient (Campbell-Tofte et al., 2011).

#### 2.2.2 Tuckwheat yuzhu tea

Tuckwheat yuzhu tea is composed of *Fagopyrum tataricum* (L.) Gaertn. seeds and dried rhizomes of *Polygonatum odoratum* (Mill.) Druce. It is commonly used as a hypoglycemic drug in Traditional Chinese Medicine (TCM). In an RCT, the FPG level decreased significantly in the intervention group of middle-aged T2DM patients; however, the HbA1c level did not change significantly. This may be due to the short observation period (Yue et al., 2018).

#### 2.2.3 Mulberry leaf black tea

With the gradual confirmation of the hypoglycemic function of mulberry leaves, various HTs containing mulberry leaves have been developed and used. In China, many people know HTs, such as Mulberry leaf black tea, Mulberry leaf Sophora japonica tea, and Mulberry leaf oolong tea. However, the hypoglycemic effect of these HTs has rarely been confirmed in clinical studies. Currently, only one RCT has confirmed the hypoglycemic effect of mulberry leaf black tea in T2DM patients. However, after 12 weeks of treatment, FPG, HbA1C, and OGTT2h levels decreased more in the treatment group than in the control group (Liu Zhan-Liang et al., 2018).

##### 2.2.4 Shuning tea

Shuning tea, native to China, is a mixture of various herbs. Mulberry leaves are the main ingredients. In a 1-month RCT, the improvement in blood glucose levels was better in the Shu Ning tea combined with the metformin group than in the metformin-only group (Fang, 2001).

##### 2.2.5 Zhongyue Jiangsangao tea

Zhongyue Jiangsangao tea consists of 10 kinds of Chinese medicines including hawthorn. After 3 months of treatment with this HT, Han found that FPG, 2hPBG, fasting insulin, and HOMA-IR of patients with T2DM diagnosed for the first time were significantly lower than those in the control group (Chao-yang, 2015).

##### 2.2.6 Tianhan Xiaoke tea

Tianhan Xiaoke (THXK) tea contains various traditional Chinese medicines and green tea, developed by the Shaanxi Tea Research Institute in China. An RCT showed that after taking THXK tea for 2 months, the decreased range of FPG and HbA1C in the observation group was significantly different from that in the control group (Hu et al., 2018).

##### 2.2.7 Diabetes tea

The main ingredient in Diabetes tea is black tea derived from *Camellia sinensis* (L.) Kuntze. It is supplemented by 12 other medicinal plants. Patients were administered three cups (600 ml) of diabetes tea extract or placebo extract daily for 12 weeks. At the end of the study period, a significant decrease in HbA1c levels was observed in the diabetes tea group. In addition, a study also showed that diabetes tea has anti-inflammatory and anti-hyperlipidemic effects (Mahmoud et al., 2016).

##### 2.2.8 Self-made Chinese herbal tea

Wen et al. developed an HT consisting of 15 g of mulberry leaves, 15 g of hawthorn, 15 g of lotus leaves, and 15 g of green tea. In a 6-months RCT, the control group used acupressure intervention, and the experimental group used HT. At the end of the experiment, the decrease in HbA1c, FPG, and oral glucose tolerance test (OGTT) levels were significantly greater in the experimental group than in the control group (Wen and Xuejun, 2018).

In conclusion, 16 RCTs confirmed the benefits of HTs in improving blood sugar in T2DM patients (Table 1). Among them, six kinds of medicinal tea, including mulberry leaf tea, produce the main hypoglycemic effect as a single herbal medicine. In addition, these seven kinds of HTs, including Rauvolfia-Citrus tea, play a role in lowering blood sugar levels through various herbs. We have listed HTs’ doses and administration methods in Table 3 to improve their clinical use further. RC tea was prepared by boiling Rauvolfia vomitoria foliage and Citrus aurantium fruits (400 g: 2 kg for 10 L). It was administered in 250 ml portions taken three times daily with the three main meals of the day (Campbell-Tofte et al., 2011). The remaining HTs were brewed by the patients. In addition, we evaluated the quality of the included RCTs using the modified Jadad rating scale. Nine articles scored 1-3. Seven articles scored 4-7. Overall, the quality of the articles was low.

## 3 Polyphenolic compounds

Polyphenols have long been considered the main reason for tea’s wide range of health benefits (Khan and Mukhtar, 2018). Polyphenols typically contain at least one or more aromatic rings linked to hydroxyl groups. There is growing evidence that a moderate long-term intake of this substance can benefit the incidence of cancer and chronic diseases (Cao et al., 2019; Fraga et al., 2019). Epidemiological findings have shown that plant polyphenols can manage and prevent T2DM. Table 4 shows herbs’ total polyphenol and polyphenol content in the first category of HTs (Kubola and Siriamornpun, 2008; Mateos et al., 2018; Juan, 2019; Catani et al., 2021; Zhen et al., 2021). The polyphenol content in herbs of different origins differs. The table shows the content of other phenolic compounds in the herbs in the category with the highest total polyphenol content. Currently, studies on the polyphenol content of Puda tea are lacking; therefore, only the polyphenol content of its main component, bitter melon, is shown. Because of the complexity of the composition of the second group of HTs and the synergistic effects between various compounds, their polyphenol contents are not discussed for now.

**TABLE 4:** Polyphenol content in herbs.

HTs	Total polyphenols (mg/g)	Content of polyphenolic compounds (mg/g)							References
Mulberry leaf tea	13.36	Astragalin	Chlorogenic acid	Kaempferol-glycoside	Quercetin-glycoside	Quercetin	Rutinum	-	Deng et al. (2021)
		0.09	1.106	0.434	0.502	1.099	0.814	-	
Chamomile tea	100.5	Apigenin	Apigenin-7-O-glucoside	Chlorogenic acid	Caffeic acid	Ferulic acid	Luteolin	Luteolin-7-O-glucoside	Catani MV et al. (2021)
		1.22	9.93	13.25	0.77	2.85	2.6	6.51	
		p-coumaric acid	Quercetin	Rutinum	-	-	-	-	
		10.05	1.97	1.58	-	-	-	-	
Gynostemma tea	75	Isoquercitrin	Kaempferol	Para-hydroxybenzoic acid	Protocatechuic acid	Quercetin	Rutinum	-	Deng et al. (2019)
		0.36	0.82	0.04	0.15	1.36	10.07	-	
Yerba mate tea	84.887	Caffeic acid	Caffeoyl-glycosides	Caffeoylquinic acids	Caffeoyl-feruloylquinic acids	Caffeoyl-p-coumaroylquinic acids	Caffeoyl-sinapoylquinic acids	Dicaffeoylquinic acids	Mateos R et al. (2018)
		0.231	2.9	54.134	0.548	0.053	0.091	16.608	
		Feruloylquinic acids	Kaempferol-glycoside	Kaempferol-rhamnoglucoside	p-Coumaroylquinic acids	Quercetin-glycoside	Rutinum	-	
		2.033	7.604	0.922	0.358	0.581	0.581	-	
Bitter melon	224	Catechin	Gallic acid	p-Coumaric	Tannic acid	-	-	-	Kubola and Siriamornpun (2008)
		4.54*	202*	0.16*	1.41*	-	-	-	

In this table, the total polyphenol content was determined by Folin-Ciocalteu colorimetric method and the phenolic compounds were analyzed by high performance liquid chromatography.

*The units of these values are mg/l.

## 4 Safety assessment

Currently, HT is widely used worldwide; however, owing to the lack of professional guidance, its security remains the focus of attention. Li et al. evaluated the toxicological profile of mulberry leaf extract (MLE) using acute, subacute toxicity, and genotoxicity tests. They considered MLE safe, supporting its application as a novel food ingredient or product (Li et al., 2018). The safety of KT root water extract was evaluated using DNA microarray, and the results showed that KT tea had no acute liver toxicity (Im et al., 2008). In addition, reverse mutation, chromosome aberration, and mouse micronucleus assays were used to evaluate the potential genotoxicity of KT root extract. The study results indicated that the plant is safe (Flammang et al., 2006).

Notably, some HTs have certain contraindications. There have been two cases of hypoglycemia and convulsion in children after taking balsam pear tea (Raman and Lau, 1996). The use of balsam pear tea in children should be avoided because of the lack of dose advice. In addition, couples preparing for pregnancy should carefully consider the intake of bitter gourd because it has shown significant characteristics of inhibiting sperm motility and promoting abortion (Basch et al., 2003). Individuals with G6P dehydrogenase deficiency and renal insufficiency should also avoid large doses or prolonged use of bitter melon. Significant adverse reactions have been reported after taking Momordica charantia in these two groups of patients (Raman and Lau, 1996). Patients with severe allergic reactions to chamomile and a history of contact dermatitis should avoid chamomile tea consumption. In addition, coumarin in chamomile can enhance the therapeutic effect of warfarin and provide additional hemodilution of aspirin and other drugs (Heck et al., 2000; Abebe, 2002). Therefore, chamomile tea should be avoided with the above two drugs. Because chamomile has a mild sedative effect, it can also increase the effect of other sedative drugs, such as apioid analgesics, **benzodiazepines**, or alcohol, on the central nervous system (McKay and Blumberg, 2006; Nguyen et al., 2021). Therefore, the author suggests that Chamomile tea use should be minimized after consuming these drugs and drinking much alcohol. In a recent toxicity study, GP showed good safety. However, Gynostemma can increase the toxicity of other drugs. Therefore, when used in combination with other drugs, drinkers’ liver and kidney function should be watched. A study on YM tea showed that drinking beverages had been linked to an increased risk of cancer (Bracesco et al., 2011). The polycyclic aromatic hydrocarbon content of YM tea may cause an increased incidence of cancer (Kamangar et al., 2008; Bracesco et al., 2011). In addition, hot water brewing may also be the reason for an increased incidence of cancer, particularly esophageal cancer (Humans IWGotEoCRt, 2018). Furthermore, a clinical study conducted in Uruguay between 1990 and 2004 supplemented the carcinogenic characteristics of hot YM teas. The study’s findings showed that drinking hot YM tea was strongly associated with cancers of the esophagus, lung, and bladder and was significantly associated with cancers of the cervix, prostate, and kidney. However, no correlation was observed for gastric, colon, rectal, and breast cancers (Stefani et al., 2011).

In conclusion, these HTs should be consumed daily under the guidance of medical professionals. If necessary, the number of hypoglycemic drugs and insulin should be adjusted over time. In addition, because a doctor’s permission is not required to obtain HTs, we should be wary of their abuse.

## 5 Mechanism research

**FIGURE 1:**
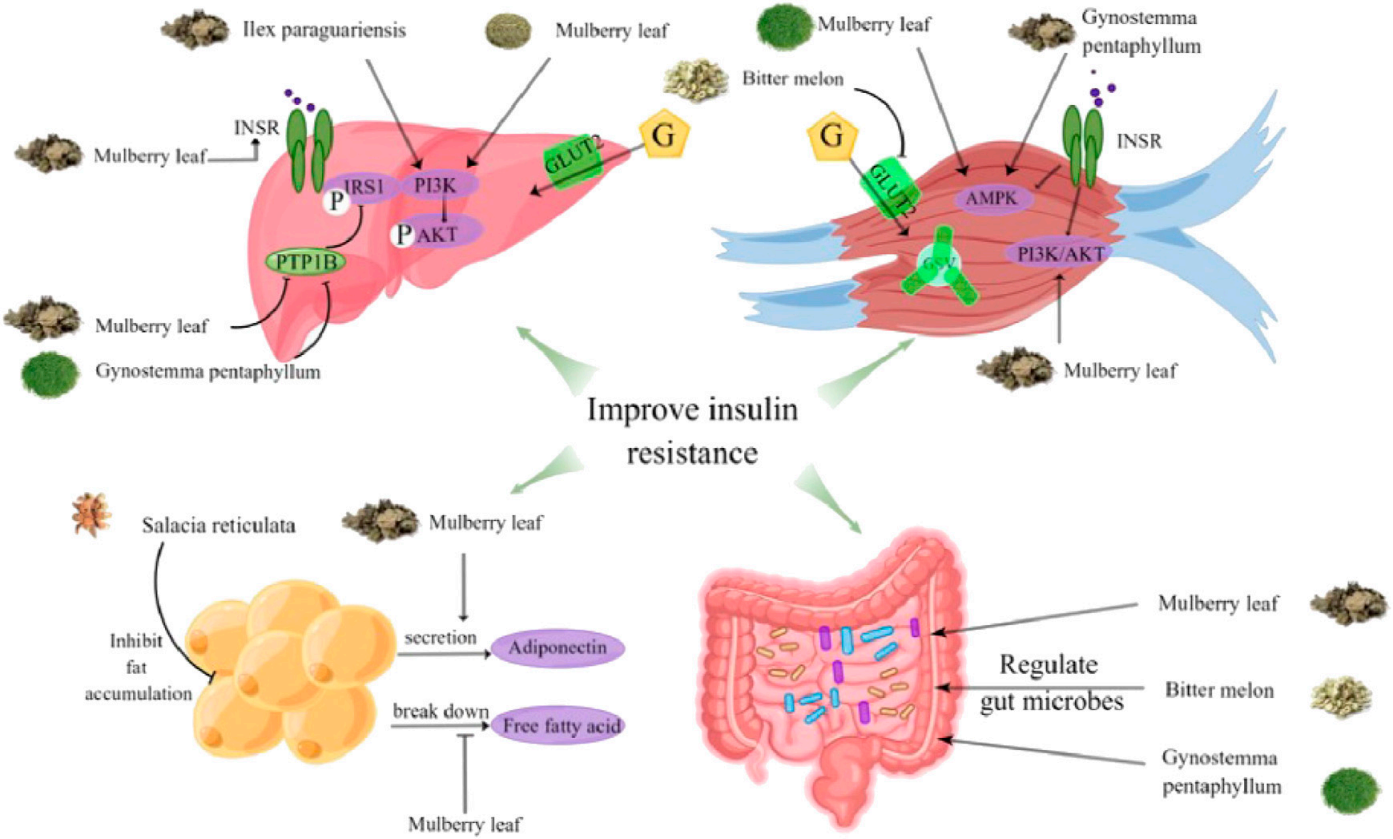
The effects of herbal teas in improving insulin resistance.

**FIGURE 2:**
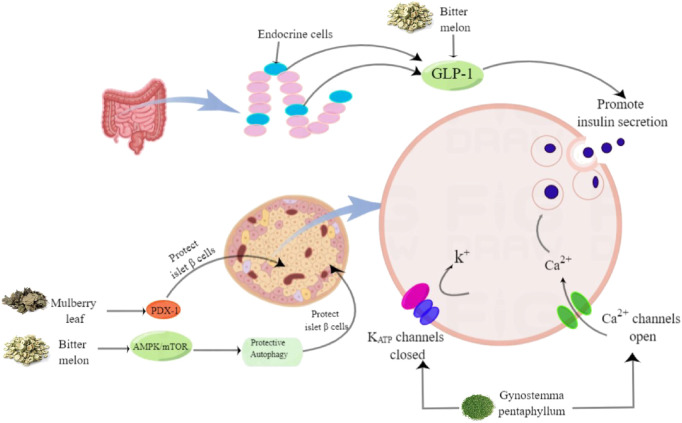
The effects of herbal teas in protecting islet beta cells and promoting insulin secretion.

**FIGURE 3:**
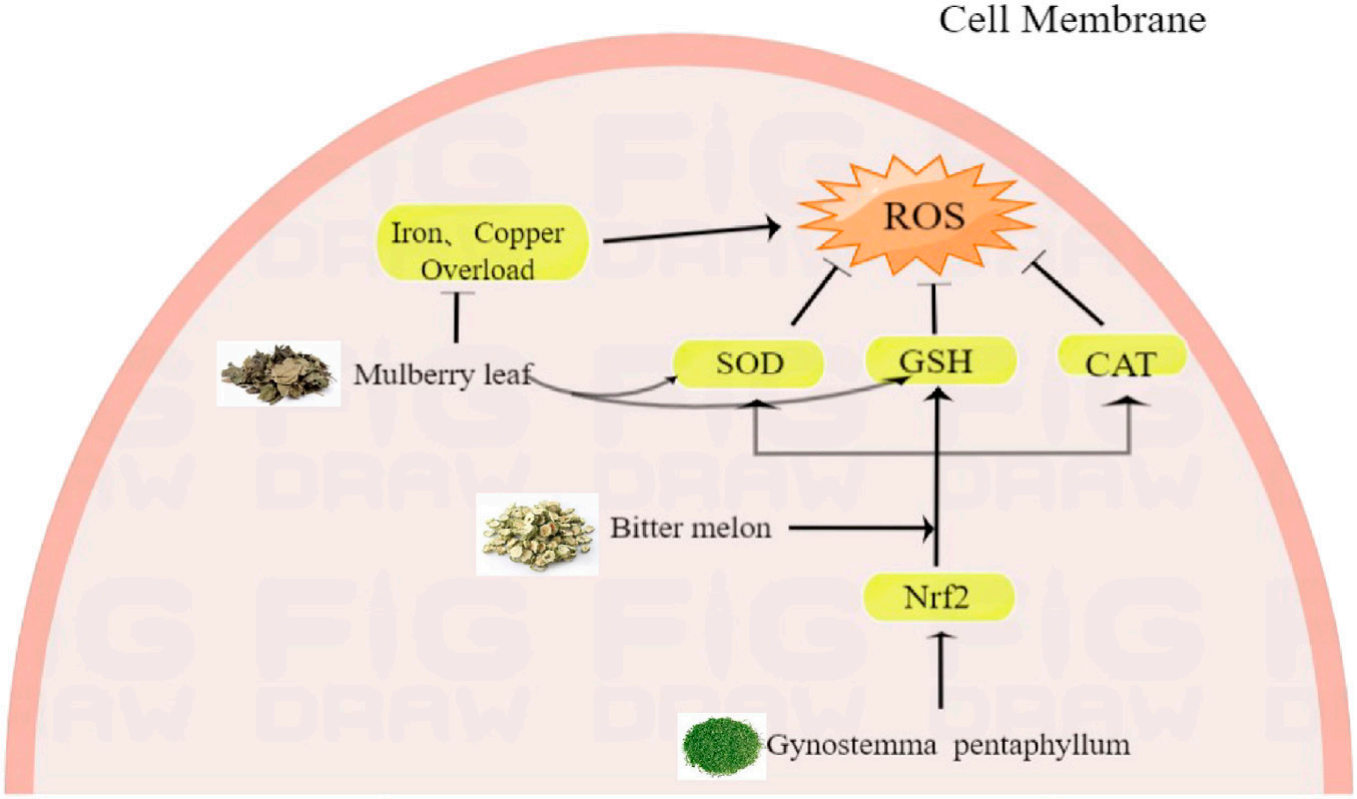
The Anti-inflammatory and anti-oxidant effect of herbal teas in Type 2 diabetes mellitus.

**FIGURE 4:**
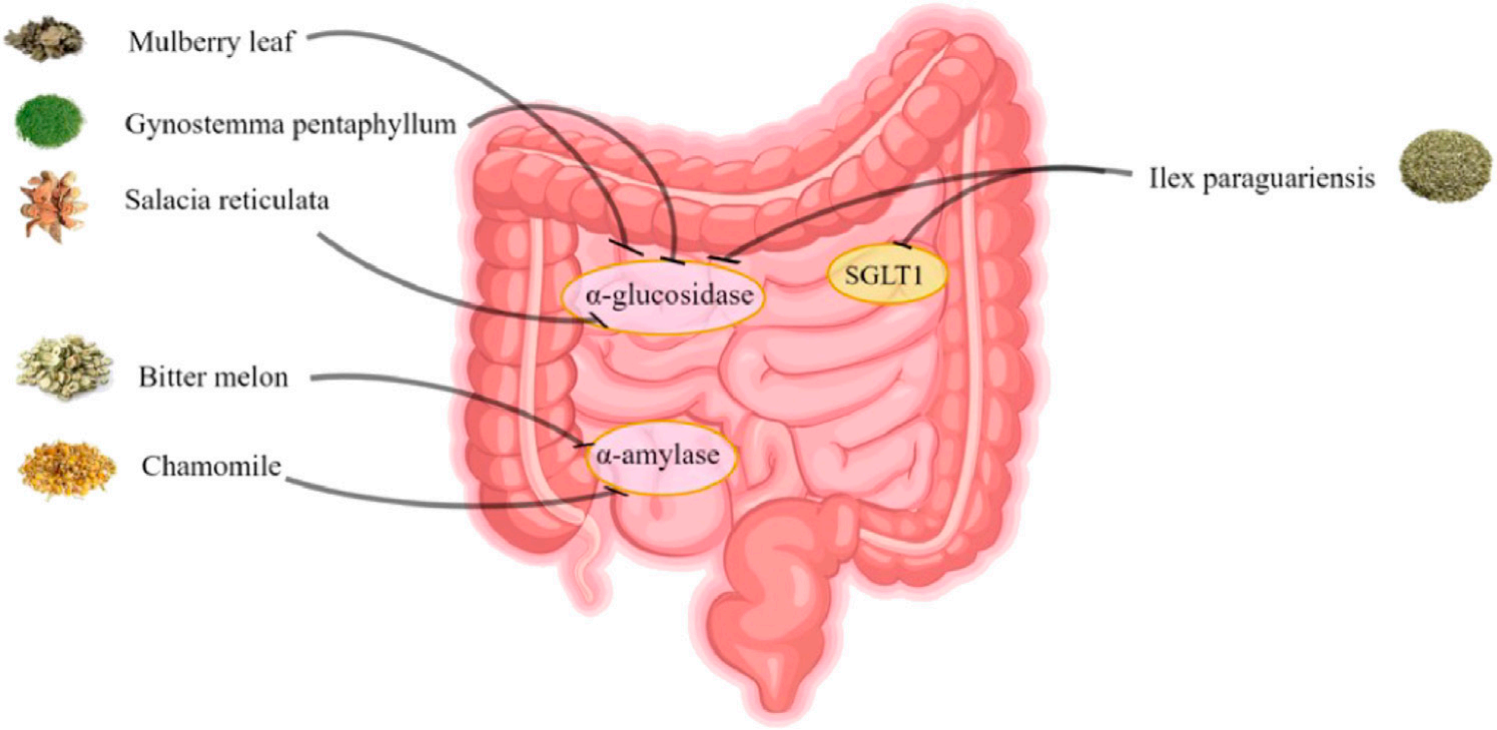
Inhibitory effect of herbal teas on glucose absorption.

**FIGURE 5:**
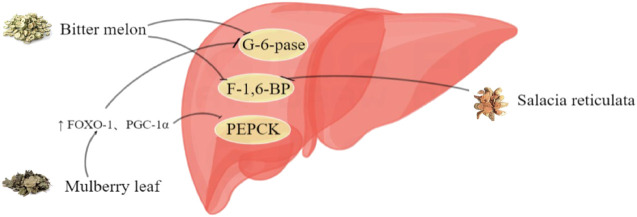
Inhibitory effect of herbal teas on gluconeogenesis.

**FIGURE 6:**
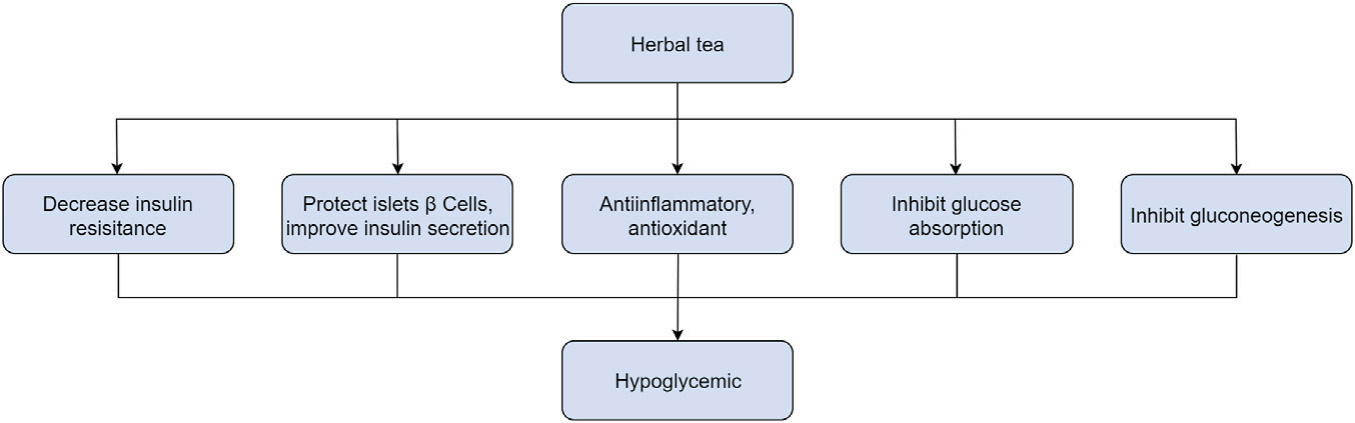
The effect of herbal teas in type 2 diabetes mellitus.

In RCTs, 14 HTs have been shown to have antidiabetic activity. However, the hypoglycemic mechanism of HTs composed of multiple herbs is complex, and the interactions between drugs are unclear. Therefore, this section only comprehensively discusses the mechanism of action of HTs composed of single herbs (Figures 1–6, Table 5).

**TABLE 5:** Hypoglycemic mechanism of herbal tea based on single herbal tea.

Herbal Tea Name	Extract	Animal model/ Cell	Duration	Effect	In vivo/vitro	References
Mulberry leaf tea	Mulberry leaf polysaccharide (MLPII)	Male Wistar rats; High-fat diet; injection of low-dose STZ	6 weeks	Inhibiting the expression of PTP1B, activating the PI3K–AKT pathway and mitigating oxidative stress	*in vivo*	Ren et al. (2015)
	Mulberry leaf	Male Sprague-Dawley rats; High-fat diet; injection of low-dose STZ	13 weeks	inhibiting NEFA signaling pathway, improving the community structure of the intestinal microbiota	*in vivo*	Sheng et al. (2017)
	Water extracts of mulberry leaf (WEM)	Sprague-Dawley male rats; high-fat and high-sugar diet; injection of low-dose STZ	10 weeks	Inhibiting TLR2 signalling pathway, Stimulation of insulin signal pathway, Inhibit the production of TNF- α in serum	*in vivo*	Tian et al. (2019a)
	mulberry leaf extract (MLE)	Male db/db mice	8 weeks	stimulating glucose disposal in skeletal muscle cells via the PI3K/Akt and AMPK pathways	*in vivo*	Ui-Jin Bae, et al. (2018)
	Mulberry leaf flavonoids (MLF)	Male db/db mice and db/m mice	7 weeks	Ameliorating muscle glucose uptake and mitochondrial function in L6 muscle cells via AMPK/PGC/1α signaling pathway	*in vivo*	Qinghai Meng, et al. (2020)
	Folium Mori extract (FME): contained the flavonoid and polyphenol components	Male Sprague-Dawley rats; high fat and high sugar diet; injection of STZ	4 weeks	Activating the IRS-1/PI3K/Glut4 signalling pathway in skeletal muscles	*in vivo*	Shengyu Cai, et al. (2016)
	Mulberry leaf extract (MA)	Mouse 3T3-L1 preadipocytes	8 days	Stimulating adipogenesis and adiponectin secretion in 3T3-L1 cells	*in vitro*	Naowaboot et al. (2012)
	Mulberry anthocyanin extract (MAE)	Male db/db mice and their nondiabetic lean littermates	8 weeks	Activating of PI3K/AKT pathways, activating AKT phosphorylation and its downstream targets in insulin-sensitive tissues	*in vivo*	Fujie Yan, et al. (2016)
	Mulberry leaf	Male Sprague-Dawley rats; High-fat diet; injection of low-dose STZ	13 weks	Inhibition of NEFA signalling pathway transduction, restored the phyla Bacteroidetes and Proteobacteria and class Clostridia in the intestinal tract	*in vivo*	Sheng et al. (2017)
	Mulberry leaf	Male db/db mice	20 weeks	Maintain insulin levels and pancreatic β-cell mass by suppressing endoplasmic reticulum stress	*in vivo*	Suthamwong P, et al. (2020)
	Mulberry leaf	Female Fischer rats; injection of alloxan monohydrate	30 days	Decreasing MMP-2 levels and SOD/CAT ratio	*in vivo*	Araujo, et al. (2015)
	Mulberry leaf extracts (acetone-water (AE) and ethanol-water (EE))	Male Wistar rats; High-fat diet; injection of STZ	4 weeks	Reducing the uptake of Fe and Cu ions and mitigating oxidative events	*in vivo*	Król et al. (2016)
	Mulberry leaf polysaccharide (MLPII)	Male Wistar rats; High-fat diet; injection of STZ	5 weeks	Inhibiting pancreatic islet apoptosis via elevation of Bcl-2/Bax ratio, ameliorating insulin secretory capacity via restoration of PDX-1 nuclear localization and expression levels	*in vivo*	Zhang et al. (2014)
	Morus alba leaves ethanol extract (MLE)	Male Sprague-Dawley (SD) rats; High-fat diet; injection of STZ	8 weeks	Protect islet cells against dysfunction and death by inducing AMPK/mTOR-mediated autophagy	*in vivo* and *in vitro*	Ji et al. (2021)
	Cryptochlorogenic acid (CCA)	Sprague-Dawley (SD) rats; injection of STZ	2 weeks	Inhibition of ferroptosis via activation of xc-/GPX4/Nrf2 and inhibition of NCOA4	*in vivo* and *in vitro*	Zhou Y. (2020)
	Mulberry leaf	High-fat mice	14 weeks	Inhibiting α-glucosidase activity; reducing the serum-free fatty acid (FFA), tumor necrosis factor-α (TNF-α), insulin, and glycated serum protein content and improving intestinal microbiota	*in vivo* and *in vitro*	Han X, et al. (2020)
	Mulberry anthocyanin extract (MAE)	HepG2 cells; male C57BL6/J genetic background (db/db) mice	8 weeks	Activating PI3K/AKT pathways	*in vivo* and *in vitro*	Yan et al. (2016)
Puda tea	Bitter melon	Male OLETF rats	6 weeks	Inhibiting NF-κB and JNK pathways	*in vivo*	Yang et al. (2015)
	M. charantia ethanol extracts (MCE)	SPF-grade male SD rats; High-fat diet; injection of STZ	8 weeks	Increase in hepatic glycogen, peripheral tissue’s GLUT-4 expression, and higher insulin sensitivity by down-regulating the expression of SOCS-3 and JNK.	*in vivo*	Ma et al. (2017)
	Bitter melon extract (BME)	NCI-H716 cells and IEC-18 cells	6 h	Activating the TAS2R-signaling pathway in enteroendocrine cells and leading to GLP-1 secretion	*in vitro*	Chang et al. (2021)
	ethyl acetate (EtOAc)-soluble fraction	Male Lepob/ob (ob/ob) and Lep+/+ (wild-type) mice	7 days	Increasing the levels of both insulin receptor mRNA and protein, decreasing the interleukin-1β mRNA and hepatic lipid accumulation in hepatocytes	*in vivo*	Dwijayanti et al. (2020)
	Bitter melon protein extract	Male Wistar rats; High-fat diet; injection of low-dose STZ	30 days	Anti-lipidemic and antioxidant	*in vivo*	Poovitha et al. (2020)
	Saponins of Momordica charantia L. (SMC)	Male Kunming mice; High-fat diet; injection of low-dose STZ	35 days	Activating AMPK/NF-κB signal pathway	*in vivo*	Wang et al. (2019b)
	polysaccharides of Momordica charantia L. (PMC)	Male Kunming mice; High-fat diet; injection of low-dose STZ	35 days	Repairing the damaged pancreatic-β cells and promoting antioxidantcapacity	*in vivo*	Wang et al. (2019a)
	Momordica charantia	Rats; injection of STZ	6 weeks	Activating pancreatic beta cells and protecting liver tissue	*in vivo*	Malekshahi et al. (2019)
	Momordica charantia fruit pulp ethanolic extract	Wistar rats, injection of STZ	28 days	Improved serum insulin and β-cell function	*in vivo*	Hafizur et al. (2011)
	Momordica charantia extracts	Male albino rats	90 min	Inhibiting of glucose-6-phosphatase and fructose-1, 6-bisphosphatase, enhancing principal enzyme G6PDH	*in vivo*	Shibib et al. (1993)
	Momordica charantia seeds	Male Wistar rats, injection of Alloxan	3 days	Contain an effective anti-hyperglycemic protein(s)	*in vivo*	Choudhary et al. (2012)
Chamomile Tea	Chamomile flowers extract (CFE)	Male C57BL/6 mice; Primary subcutaneous preadipocytes	20 weeks; 6 weeks	Activing PPARs and other factors	*in vivo* and *in vitro*	Weidner et al. (2013)
	luteolin	3T3-L1 adipocytes	24 h	Activating the PPARγ pathway and by acting at insulin signaling cascade	*in vivo*	Ding et al. (2010)
	Chamomile flowers	Male Wistar rats, injection of STZ	21 days	Inhibition of hepatic GP, inhibition of ALR2	*in vivo*	Kato et al. (2008)
	Maltodextrin-Free Chamomile	The Caco-2 cell line	4 days	Acute inhibition of GLUT2 and GLUT5	*in vitro*	Villa-Rodriguez, et al. (2018)
Gynostemma pentaphyllum Tea	polysaccharide (GPP) extracted from Gynostemma pentaphyllum herb	Kunming mice, injection STZ	4 weeks	Enhancing the SOD, CAT, and GSH-Px activities, decreasing the MDA activity, improving the levels of IL-4 and IL-10, and decreasing the levels of TNF-α and IL-6	*in vivo* and *in vitro*	Wang et al. (2020a)
	Gynostemma pentaphyllum saponins (GPs)	Male Wistar rats; injection STZ	40 days	Antioxidant effect	*in vivo*	Gao et al. (2016)
	Gynostemma pentaphyllum	Male C57 BL/6J mice, high-fat diet	12 weeks	Improve glycolipid metabolism, and stimulate BAT activity, WAT browning and lipid β-oxidation, while decreasing the ratio of Firmicutes to Bacteroidetes and enhancing the abundance of Akkermansia muciniphila	*in vivo*	Liu et al. (2017)
	Damulin A and damulin B	L6 cells		Activating AMPK, increasing β-oxidation and glucose uptake, increasing GluT4 translocation to the plasma membrane	*in vitro*	Nguyen et al. (2011)
	gypenoside	Normal Wistar rats and Diabetic Goto-Kakizaki rats		Stimulating insulin secretion	*in vivo*	Hoa et al. (2007)
	a polysaccharide (GPP)	Specific-pathogen-free mice;		Inhibiting α-glucosidase activity, inhibiting the glucose absorption, affecting the protein expression of GLUT2	*in vivo*	Wang et al. (2019a)
	Gynostemma pentaphyllum Extract	Male spontaneous type 2 diabetic Goto-Kakizaki rats		Stimulation of insulin release via K-ATP and L-type Ca2+ channels	*in vivo*	Lokman et al. (2015)
	Gynostemma pentaphyllum	Male Zucker fatty rats	5 weeks	Inhibiting alpha-glucosidase activity, improving insulin receptor sensitivity	*in vivo*	Megalli S, et al. (2006)
	G. pentaphyllum ethanol extract (GPE)	Male C57BL/KsJ-db/db mice	5 weeks	Enhancing insulin secretion and its sensitivity, enhancing hepatic glucose utilization	*in vivo*	Yeo et al. (2008)
Salacia reticulata Tea	Salacia extract	Male TSOD mice and male TSNO mice	8 weeks	Preventing obesity and associated metabolic disorders including the development of metabolic syndrome	*in vivo*	Akase et al. (2011)
	Mangiferin	Male KK-Ay mice	4 weeks	Down-regulating the gluconeogenic pathway through regulation of FBP expression	*in vivo*	Im et al. (2009)
Yerba Mate Tea	Yerba Mate	Male Wistar rats; injection of alloxan	28 days	Decreasing the intestinal SGLT1 gene expression	*in vivo*	Oliveira, et al. (2008)
	Ilex paraguariensis tea	Male Wistar rats	180 min	Inducing-insulin secretion; inhibiting *in vitro* disaccharidases activities	*in vivo* and *in vitro*	Pereira, et al. (2012)
	Yerba Mate Tea	Male Swiss strain mice; high fat diets	8 weeks	Inhibiting hepatic and muscle TNF-α and restoring hepatic insulin signalling	*in vivo*	Arçari et al. (2011)
	Gallic acid	3T3-L1 cells	30 min	Increasing GLUT4 translocation and glucose uptake activity	*in vitro*	Prasad CN, et al. (2010)

### 5.1 Improve insulin resistance

Insulin resistance (IR) refers to the decreased sensitivity of surrounding target tissues to insulin, such as the liver, muscle, and adipose tissue. IR, the main pathogenesis of T2DM, is a difficult problem treating diabetes. Therefore, improving insulin resistance during diabetes treatment is important (Petersen and Shulman, 2018).

IR is involved in a complex insulin signal transduction network. The insulin receptor (IRS) and insulin receptor substrates (IRSs) are key nodes in insulin signaling (Taniguchi et al., 2006). Recent studies have shown that the water extract of mulberry leaves can upregulate the expression of IRS and insulin receptor in the adipose tissue of diabetic mice (Tian et al., 2019a). Suppressor cytokine signaling-3 (SOCS3) is a cytokine signal transduction inhibitor family member that can significantly inhibit the activation of major signaling molecules in the insulin signaling pathway. Momordica charantia polysaccharide can improve insulin sensitivity by downregulating the expression of SOCS3 (Ma et al., 2017).

The skeletal muscle, considered the major driver of systemic insulin resistance, is responsible for approximately 80% of postprandial glucose clearance (Merz and Thurmond, 2020). In skeletal muscle, two pathways are responsible for glucose transport: the PI3K/Akt and AMPK pathways (Saltiel and Kahn, 2001). Studies have shown that MLE can improve glucose utilization in skeletal muscles using the PI3K/AKT and AMPK pathways (Bae et al., 2018).

The liver, an important organ for human energy metabolism, is a key target organ of IR in T2DM. In the liver, the PI3K/Akt pathway is responsible for most of the metabolic activity of insulin. It is activated by tyrosine phosphorylation of IRS upon insulin stimulation (Huang et al., 2018b). Tyrosine dephosphorylation is regulated by tyrosine phosphatases (PTPs). A study showed that mulberry polysaccharides (MLPII) could restore the blood glucose level in diabetic rats. This may be related to the expression of PI3K and AKT2, reducing the expression of protein tyrosine phosphatase 1B (PTP1B) (Ren et al., 2015). Similar to MLPII, dammarane-type triterpenes in GP also showed inhibitory activity against PTP1B (Hamid et al., 2015). In addition, YM tea can restore the phosphorylation of IRS1 and Akt in the liver and muscle and reduce blood glucose levels in obese mice (Arcari et al., 2011).

Dysfunctional adipose tissue plays a key role in IR (Ahmed et al., 2021). Adiponectin, an adipokine, is specifically expressed in adipose tissue and can directly reverse IR. *In vitro* studies have shown that MLE can promote the secretion of adiponectin in 3T3-L1 preadipocytes in mice (Naowaboot et al., 2012). *In vivo* studies have shown that mulberry leaves’ anthocyanin extract (MAE) can significantly increase adiponectin levels in db/db mice (Yan et al., 2016). An important factor regulating insulin sensitivity is increased free fatty acid (NEFAs) concentration, which can lead to IR through multiple mechanisms (Shulman, 2000). Sheng et al. suggested that mulberry leaves might improve IR by inhibiting NEFA signal transduction (Sheng et al., 2017). Dysfunctional obese adipose tissue can also develop IR by inducing lipotoxicity (Ahmed et al., 2021). Akase reported that Salacia reticulata extract could improve IR in mice by inhibiting visceral fat accumulation (Akase et al., 2011).

The intestinal tract is considered an important tissue for blood glucose control. Bitter gourd extract can interact with intestinal epithelial cells before circulating to other tissues. Bitter gourd ethanol extract can improve IR in intestinal cells by acting as an insulin sensitizer and replacement. Specifically, the insulin-sensitizing effect of bitter gourd may be associated with restoring insulin-induced activation of Akt signaling. Since AMPK protein mediates the insulin-independent increase in glucose, the insulin replacement function may be associated with the activation of AMPK protein (Chang et al., 2021). In addition, changes in the gut microbiota composition may be linked to the development of hyperglycemia and diabetes (Thingholm et al., 2019). Studies have shown that the structure of bitter gourd polysaccharides can be altered by fermentation with *Lactobacillus* Plantarum. This change can increase the production of single-chain fatty acids, enhancing the antidiabetic effect of balsam pear polysaccharides in rats (Gao et al., 2018). In addition, mulberry leaves may improve IR by restoring Bacteroides, Proteus, and Clostridium species in diabetic rats (Sheng et al., 2017). GP can also regulate intestinal microflora to improve IR (74).

### 5.2 Protect islet beta cells and promote insulin secretion

Autophagy is important for normal homeostasis and the survival of islet cells. Therefore, induction of islet cell-protective autophagy using drugs or other techniques is considered a promising approach for treating or preventing T2DM (Marasco and Linnemann, 2018). By inducing AMPK/mTOR pathway-mediated autophagy, the mulberry leaf ethanolic extract can protect islet cells (Liu et al., 2017). Pancreatic duodenal homeobox-1 (PDX-1), a “master regulator” of pancreatic development, is the most critical intracellular factor in beta cells. If the gene encoding this protein is missing, the human pancreas becomes underdeveloped. Malekshahi suggested that bitter gourd might have a protective effect on pancreatic beta cells by upregulating the expression of PDX-1 (Malekshahi et al., 2019). In addition, bitter gourd ethanolic extract stimulates insulin secretion by activating TAS2Rs as GLP-1 secretagogues in enteroendocrine cells (Müller et al., 2019). *In vivo*, GP extract significantly improved glucose tolerance and increased plasma insulin levels in Goto-Kakizaki rats (Hoa et al., 2007). *In vitro*, GP extract stimulated insulin release from isolated rat islets under high glucose conditions. This may be mediated by adenosine triphosphate-dependent potassium channels and L-type calcium channels (Lokman et al., 2015). In addition, the research by Pereira DF showed that the ethyl acetate and n-butanol components of YM tea also have significant insulin secretion-stimulating effects (Pereira et al., 2012).

### 5.3 Anti-inflammatory and antioxidant

Low-grade inflammation is a typical manifestation of T2DM. High glucose levels increase the production of pro-inflammatory cytokines such as tumor necrosis factor-α (TNF-α) (Hameed et al., 2015). Chronic and low-level administration of TNF-α in rodents results in systemic IR, whereas neutralization of TNF-α significantly increases insulin uptake to peripheral glucose (Lang et al., 1992; Hotamisligil et al., 1993). Wang et al. found that GP significantly reduces TNF-α levels (Wang et al., 2020a). Blocking the NF-KB signaling pathway is also an effective way to reduce the production of pro-inflammatory cytokines. In 2006, Bai et al. discovered that bitter gourd could inhibit the activation of NF-kB by inhibiting the degradation of IkBα(Yang et al., 2015).

Redox processes are important in the human body (Sies et al., 2017). Physiologically, the body is in a redox homeostasis state. When the human body is subjected to various harmful stimuli, homeostasis is disrupted, eventually damaging the body. Because the activity of various antioxidant enzymes in pancreatic islets is lower than that in other tissues, oxidative stress is most harmful to human pancreatic cells (Tiedge et al., 1997). Oxidative stress is not only considered to be responsible for the progressive dysfunction of beta cells but also a possible causative factor in the development of IR (Robertson et al., 2003; Bloch-Damti and Bashan, 2005).

Wang et al. found that bitter gourd polysaccharides can significantly improve the body’s antioxidant capacity and alleviate pancreatic damage caused by streptozotocin. The protein extract of bitter gourd can significantly increase the SOD, CAT, and GSH-Px levels, reduce glutathione (GSH) levels and improve IR (Wang et al., 2019a). Antioxidant experiments have shown that the polysaccharide components of GP can also increase the activities of SOD, CAT, and GSH-Px, reduce malondialdehyde (MDA) activity, and improve antioxidant capacity (Wang et al., 2020a). Furthermore, total Gynostemma saponins can stimulate insulin secretion by stimulating the Nrf2 antioxidant pathway (Gao et al., 2016). Studies have shown that the oral intake of mulberry leaves maintains beta-cell function and reduces pancreatic endoplasmic reticulum stress in db/db mice. MLE can effectively restore the SOD/ CAT balance in alloxan-induced diabetic rats and increase the reduced GSH/oxidized glutathione (GSSG) ratio, thereby minimizing the damage to islet beta cells caused by oxidative stress (Araujo et al., 2015). In addition, essential trace elements, especially iron, zinc, copper, and manganese, play key roles as catalytic lefts for various enzymes in various redox reactions. Both the deficiencies and excess of these micronutrients perturb the antioxidant balance, increasing free radical formation and oxidative stress in cells and tissues (Dubey et al., 2020). MLEs can correct iron and copper overload to alleviate oxidative events in diabetes (Krol et al., 2016).

### 5.4 Inhibition of glucose absorption

Starch in food needs to be hydrolyzed into dextrins and oligosaccharides under the action of α-amylase and then decomposed into glucose under the action of α-glucosidase, which is finally absorbed and utilized by small intestinal cells. This reaction process causes postprandial blood glucose to rise. Therefore, in treating T2DM, alpha-amylase and alpha-glucosidase inhibitors are effective remedies for postprandial hyperglycemia (Hossain et al., 2020).

The polyhydroxyalkaloid compound found in mulberry leaves, 1-deoxywildomycin (DNJ), is a promising α-glucosidase inhibitor (Ji et al., 2016). RCTs have demonstrated that long-term intake of DNJ-enriched MLE can improve postprandial glycemic control in individuals with impaired glucose metabolism (Asai et al., 2011). The flavonoids and alkaloids in the water extract of mulberry leaves can also significantly inhibit the activity of α-glucosidase, with inhibition rates of 86.12 ± 1.79% and 87.29 ± 1.32%, respectively (Han et al., 2020). Shivanagoudra et al. isolated seven compounds from acetone and methanol extracts of bitter gourd, all exhibiting different α-amylase and α-glucosidase inhibitory activities (Perera et al., 2021). Four classes of compounds were isolated from the water-soluble fraction of Salacia reticulata: kotalanol, salacinol, 13-membered ring thiocyclitol, and 13-membered sulfoxide. These four substances are natural and effective α-glucosidase inhibitors (Liu et al., 2006; Han et al., 2020). Morikawa et al. found that the methanol fraction of Salacia reticulata Wight has an inhibitory effect on α-glucosidase (Morikawa et al., 2021). Recent research suggests that α-glucosidase inhibitors reversibly bind to maltase-glucoamylase (MGAM) and sucrase-isomaltase (SI), thereby inhibiting carbohydrate hydrolysis in the small intestine. ntMGAM is the catalytic domain of MGAM. Sim et al. believe that deoxy-sulfomatrine, an extract of Salacia reticulata, is the most potent ntMGAM inhibitor reported to date. Its inhibitory effect is approximately 2,000 times stronger than that of acarbose (Sim et al., 2010). Chamomile showed a strong inhibitory effect on MGAM and SI in brush border membranes prepared from the small intestine of rats (Kato et al., 2008). Villa-Rodriguez et al. believed that the presence of apigenin and apigenin 7-O-glucoside is one of the reasons why chamomile inhibits alpha-amylase. Unlike acarbose, this combination of polyphenols gently impairs carbohydrate digestion and sugar absorption, thereby avoiding gastrointestinal side effects (Villa-Rodriguez et al., 2018). GP’s polysaccharide components and total saponins can effectively inhibit α-glucosidase activity (Wang et al., 2019b). In addition, studies have confirmed that YM significantly inhibits disaccharidase *in vitro*, and the inhibitory effect is acute (Pereira et al., 2012).

SGLT1 plays an important role in intestinal glucose absorption. Inhibition of SGLT1 in the intestine delays and attenuates intestinal glucose absorption and prevents a rapid increase in postprandial glucose levels (Tsimihodimos et al., 2018). In a study by Oliveira, Yerba Mate significantly reduced SGLT1 gene expression in mice’s upper and middle intestines. This suggests that the bioactive compounds in Yerba mate may interfere with glucose absorption by reducing SGLT1 expression (Oliveira et al., 2008).

### 5.5 Inhibition of gluconeogenesis

It is well known that gluconeogenesis, an important part of glucose metabolism, is the main cause of increased fasting blood glucose in patients with T2DM (Petersen et al., 2017). Most HTs in this study may inhibit gluconeogenesis by inhibiting glucose-6-phosphatase (G6Pase) and fructose 1,6-bisphosphatase (FBPase) (Méndez-Lucas et al., 2013). In HepG2 cells with IR, mulberry leaf anthocyanin extract promotes the upregulation of FOXO-1 and PGC-1α, thereby reducing the activities of phosphoenolpyruvate carboxykinase (PEPCK) and G6Pase (Yan et al., 2016). Mangiferin, an aqueous extract of Salacia reticulata, acts directly on hepatocytes and inhibits FBPase expression (Im et al., 2009). A study showed that the liver glycogen content was significantly increased by approximately 75% after oral administration of the n-butanol component of YM for 3 h compared with the control group (Pereira et al., 2012). In an STZ-induced diabetic rat model, quercetin, luteolin, and apigenin in chamomile showed good inhibitory activity on glycogen phosphorylase (Kim and Jobin, 2005). In addition, genistein in mulberry leaves may ameliorate hepatic gluconeogenesis dysfunction by modulating AMPK-CRTC2 and MEK/ERK signaling pathways (Bae et al., 2018).

## 6 Discussion

T2DM is a chronic metabolic disease requiring long-term medication. The World Health Organization’s traditional medicine strategy promotes using herbal medicines to treat chronic diseases (Kishino et al., 2009). HT is an invaluable resource for in-depth studies as a special method of consuming herbs. It has the advantage of regulating multiple targets and affecting multiple biological processes. By summarizing RCTs, we found that HTs have a certain clinical efficacy in improving blood sugar levels, which can be considered a promising auxiliary hypoglycemic method for T2DM.

Compared with traditional teas, HTs are more advantageous in improving blood sugar in T2DM patients. Whether herbal medicines and teas have adjunctive therapeutic effects on T2DM is a topic of widespread concern. Liu et al. conducted a meta-analysis on the hypoglycemic effect of herbal medicines in 2004 and 2011, respectively. The results suggest that herbal medicines can improve blood sugar levels in T2DM patients (Liu et al., 2004a; Liu et al., 2004b). In 2019, Tian et al. selected high-quality RCTs published in the past 10 years for research and confirmed the efficacy of herbal medicine in improving blood sugar (Tian et al., 2019b). In addition, studies on various herbal extracts such as ginsenosides, resveratrol, and guava have shown that herbal medicines have a positive therapeutic effect on T2DM (Luo et al., 2019; Zhou et al., 2019; Huang et al., 2020). As the second most popular beverage globally, tea consumption is inversely linearly associated with the incidence of T2DM. Multiple epidemiological studies suggest that drinking four or more cups of tea daily may reduce the incidence of T2DM (Jing et al., 2009; van Woudenbergh et al., 2012). However, there is no direct evidence that tea can control blood sugar levels in T2DM patients (Li et al., 2016; Yu et al., 2017). Therefore, further exploration of whether HTs can combine the advantages of herbs and teas to play an ideal role in blood sugar control in T2DM is required.

Compared with traditional herbal decoctions, HTs are more convenient. Traditional Chinese medicine decoctions must be continuously boiled for at least 30 min, whereas HT requires only hot or cold water to brew. In addition, Chinese scholars have compared the efficacy and dissolution quality of HTs and traditional herbal decoctions. In 2003, Liu compared the differences in dissolution quality between tea preparations and traditional decoctions of four herbs. The four herbs were peony bark, rhubarb, honeysuckle, and pale bamboo leaves. The experimental results showed that the differences in dissolution quality between HT preparations and traditional decoctions of these four herbs were not statistically significant (Guangxi, 2003). Subsequently, Li et al. expanded their study to 18 herbs. They believed that herbs containing tannins and acids or fruits and leaves should be made into teas, while herbs containing volatile oils should be made into decoctions (LI Jian-kai et al., 2004). These studies suggest that HTs composed of single-flavored plants may be no less effective than traditional decoctions, except those containing volatile oils.

Whether HTs have significant sensory drawbacks is critical for consumers to remain enthusiastic about products. The ideal product should be sweet, mild, fruity, not too bitter, astringent, pungent, strong, or unfermented in flavor (Yang and Lee, 2020). Among these, astringency is the most troublesome attribute. Fresh tea leaves often contain varying amounts of astringent and bitter compounds that emit little to no aromas (Chao-hua, 2014). However, processing leads to the development of rich flavor compounds. Similarly, when mulberry leaf tea is fried, green astringency is significantly reduced, and the aroma is strong and mellow. In addition, consumers who do not tolerate the bitter taste of meta-tea and bitter melon often add sugar, juice, or honey to increase their appreciation (Samoggia et al., 2021). However, this approach does not apply to patients with diabetes. Furthermore, not all HTs have unacceptable tastes. For example, the citrus aroma in Rauvolfia-Citrus tea can somewhat offset bitterness discomfort (Campbell-Tofte et al., 2011). A systematic review of the literature on Rauvolfia-Citrus tea reveals that the HT has “fruity,” “floral,” “green leaf,” and “fatty " aromatic notes (Wang et al., 2020b). In addition, chamomile tea has been widely consumed for centuries owing to its pleasant flavor (Guzelmeric et al., 2015).

The molecular mechanism of HT in improving blood sugar involves improving IR, protecting islet β cells, anti-inflammatory, and antioxidant effects, inhibiting glucose absorption, and inhibiting gluconeogenesis. Among these, the most significant hypoglycemic mechanism of HT is improving IR. The pathogenesis of IR is a complex process. According to the different stages of pathogenesis, the pathological mechanisms can be divided into three stages: pre-receptor, receptor, and post-receptor. Abnormalities in the post-receptor link and disturbances in signal transduction are the main causes of IR (Chen et al., 2019). Similarly, improving post-receptor link abnormalities is the main method for improving IR in medicinal tea. The signaling pathways regulated by medicinal tea mainly include the PI3K/AKT, AMPK, and GLUT4 signaling pathways. In addition, based on the upregulation of insulin receptor expression by mulberry leaves and bitter gourd, we believe that the receptor link may e a way medicinal tea can improve IR. The protective effect of HTs on islet β-cells is mainly by promoting the proliferation of islet β-cells, increasing the level of GLP-1, and regulating the level of autophagy. In addition, some researchers have suggested that islet microcirculation is involved in islet β-cell dysfunction. In pre-diabetes, the islet microcirculation is in a state of hyperperfusion. This state leads to high pressure in islet microvessels and damage to endothelial cells, eventually leading to insufficient perfusion of the islet microcirculation and aggravating islet dysfunction. However, no studies have confirmed the effects of HT on islet microcirculation (xin, 2008). Notably, HTs still have anti-inflammatory and antioxidant properties similar to traditional teas. These properties are an important reason for HTs’ widespread use in managing diabetes. It is well known that traditional teas’ anti-inflammatory and antioxidant properties are attributed to their rich polyphenol content (Hussain et al., 2016). The polyphenols in tea include EGCG, quercetin, and theaflavin (Winiarska-Mieczan et al., 2021). In this study, polyphenolic compounds were the main material basis for HTs’ anti-inflammatory and antioxidant properties. Unique flavonoids in Rooibos tea, Aspalathin and Xanthone mangiferin in Honeybush tea (Ajuwon et al., 2018), and chlorogenic and gallic acids in YM tea (Oliveira et al., 2008) are all anti-inflammatory and antioxidant. In addition, herbs’ polysaccharides, saponins, and alkaloids are also the main components responsible for their anti-inflammatory and antioxidant effects (Sun et al., 2014; Chen et al., 2017; Zhang et al., 2019; Zhou et al., 2019; Song et al., 2020). In addition, HTs affect glucose metabolism by inhibiting glucose absorption and gluconeogenesis. Intestinal glucose absorption is dependent on α-glucosidase activity (Van de Laar et al., 2005). The only marketed α-glucosidase inhibitor, acarbose, have adverse gastrointestinal side effects (Chiasson et al., 2002). HTs not only inhibit α-glucosidase but also do not cause adverse reactions in the gastrointestinal tract. Therefore, it is a reliable hydrolase inhibitor Table 5.

Whether these mechanisms are linked to the actions of HTs *in vivo* requires further investigation. The HTs in this study exerted hypoglycemic effects mainly through polyphenols, polysaccharides, saponins, and alkaloids. Polyphenols are poorly absorbed in the body’s circulation either because of their low intrinsic activity or poor absorption or rapid elimination in the intestine (Manach et al., 2004). Polysaccharides originating from TCM are mostly heteropolysaccharides composed of different monosaccharides, which is why they are less stable and less absorbed (Wang et al., 2022). Saponins and alkaloids have undesirable physicochemical properties and poor pharmacokinetic characteristics (Zheng et al., 2018b; Kim et al., 2018). This study showed that the bioavailability of HTs was unclear or low. This differs from the performance of HTs reported in clinical studies. Therefore, using bioavailability to explain the beneficial hypoglycemic effects of HTs seems inappropriate. The concept of bioavailability integrates numerous variables, such as intestinal absorption, microbiota metabolism, intestinal and hepatic metabolism, and plasma kinetics (Bhattaram et al., 2002). Few studies have been conducted on HTs to integrate sufficient information and link variables to organ-level health effects. The relative weight of each bioavailability variable may depend on the specific ingredients under consideration. For example, some polyphenols may be absorbed at lower rates than others but still achieve comparable plasma concentrations due to their lower secretion into the intestinal lumen and lower metabolism and elimination (Manach et al., 2004). Therefore, there is a need to develop an accurate method to detect the specific content of hypoglycemic components in HTs and to invest more effort in determining the correlation between hypoglycemic components and their biochemical endpoints.

In this study, we suggest for the first time that HTs may be an adjunctive treatment modality to improve blood sugar levels in patients with T2DM. This may be related to the potential pharmacological effects of compounds such as polyphenols in HTS to improve blood glucose levels. The antidiabetic effects of HTs appear to be mediated by mechanisms such as improving IR, protecting islet beta cells, anti-inflammatory, and antioxidant effects, inhibiting glucose absorption, and inhibiting gluconeogenesis. While HTs have attracted the attention of researchers in recent years for their health-promoting effects, their mechanisms of action remain unclear because of the complex compounds typically present in each HT. In the future, more research is needed to evaluate the antidiabetic benefits of multiple classes of compounds in HTs to elucidate their exact mechanisms. Understanding the dosage and administration of HTs and the bioavailability of their active compounds is a prerequisite for understanding their pharmacological mechanisms of action in diabetes treatment. In addition, studies on the metabolism of bioactive compounds in HTs are needed to make effective recommendations on HT intake. However, most clinical studies have been of low quality. Therefore, there is an urgent need to design more rigorous RCTs with larger sample sizes. In conclusion, this review provides useful data and information for further research and applications of HTs in the treatment of T2DM.
